# Autistic traits in adults who have attempted suicide

**DOI:** 10.1186/s13229-019-0274-4

**Published:** 2019-06-07

**Authors:** Gareth Richards, Rebecca Kenny, Sarah Griffiths, Carrie Allison, David Mosse, Rosemary Holt, Rory C. O’Connor, Sarah Cassidy, Simon Baron-Cohen

**Affiliations:** 1 Autism Research Centre, Department of Psychiatry, Douglas House, University of Cambridge, 18b Trumpington Road, Cambridge, CB2 8AH UK; 2 School of Psychology, Newcastle University, Newcastle upon Tyne, UK; 3 SOAS, University of London, London, UK; 4 Suicidal Behaviour Research Laboratory, Institute of Health and Wellbeing, University of Glasgow, Glasgow, UK; 5 School of Psychology, University of Nottingham, Nottingham, UK; 6 CLASS Clinic, Cambridgeshire and Peterborough Foundation NHS Trust, Cambridge, UK

**Keywords:** ASC, ASD, Asperger syndrome, Autism, Autism spectrum, Autistic traits, Depression, Mental health, Suicidality, Suicide

## Abstract

**Background:**

An emerging literature suggests that autistic adults are at increased risk of experiencing suicidal thoughts, making suicidal plans and attempts, and dying by suicide. However, few studies have investigated whether autistic traits are related to suicidal behaviour. The current study examined autistic traits in a sample of adults who reported at least one suicide attempt.

**Methods:**

An online questionnaire was advertised between June and September 2017 on suicide prevention websites, research databases, and social media. Participants reported whether they had ever attempted suicide (yes/no), and if so, how many times they had attempted (once/more than once). They also reported diagnosed and suspected mental health or neurodevelopmental conditions, and completed the Autism Spectrum Quotient (AQ). Two hundred forty-five adults accessed the survey; 132 reported having attempted suicide and also completed the AQ. It was hypothesised that AQ total scores and subscale scores would be higher in adults who had attempted suicide more than once compared to adults who had attempted once. These hypotheses were tested using an independent samples *t* test, Mann-Whitney *U* tests, and binary logistic regression.

**Results:**

Most participants were female (83.3%, male = 12.9%, other = 3.8%), and ages ranged from 18 to 65 (median = 36.00; IQR = 19.00). Total AQ scores, as well as communication and imagination subscale scores were significantly higher in adults who had attempted suicide more than once compared to adults who had attempted suicide once. Even after removing participants with diagnosed or suspected autism (*n* = 34), 40.6% had an AQ score indicative of clinical concern (≥ 26).

**Conclusions:**

The findings suggest that high levels of autistic traits may frequently be present in adults who have attempted suicide, and that AQ scores are higher in those with a history of more than one suicide attempt. It may be possible to better identify suicide risk by screening autistic adults with mental health conditions for suicidal thoughts and behaviours, and by screening people with suicidal thoughts and/or behaviours for autism.

## Background

The World Health Organisation [1] estimates that approximately 800,000 people take their own lives each year, and that for every person who dies by suicide, there are many others who attempt suicide. Mortality from suicide is typically higher in males [2], whereas suicidal ideation and suicidal behaviour are more common in females [3–5]. To reduce suicide rates, it is important to identify more specific markers of risk, not only in patient groups, but also within the general population. Although the pathway to suicide is complex, recent research has highlighted a key role for psychological factors [6]. For example, aspects of personality, such as perfectionism [7], neuroticism, hopelessness, and introversion [8, 9] are associated with suicidal thoughts and behaviours [6]. Some personality constructs have also been shown to be predictive of suicidal behaviour over the life-span. Pickles et al. [10] reported that adolescent worry and irritability predict mid-life suicidality as well as mid-life affective disorders and neuroticism.

There is a growing body of research suggesting that autistic individuals are at increased risk of suicidal ideation, plans and attempts, and of dying by suicide [11–19]. Autism spectrum conditions (hereafter autism) are characterised by difficulties in communication and social reciprocal interaction, difficulties in adjusting to unexpected change, alongside unusually narrow interests and repetitive behaviour, and sensory hyper-sensitivity [20]. Cassidy et al. [11] reported data from a clinical cohort study of 374 adults (256 males, 118 females) who had recently been diagnosed with Asperger syndrome. Each had been screened for suicidal thoughts, plans and attempts prior to their diagnostic appointment. Sixty-six percent of responders reported having experienced suicidal thoughts, and 35% reported that they had made plans or attempts at suicide. The rate of suicidal ideation in this sample was significantly higher than that of the general UK population, people with one, two, or more medical illnesses, and people with psychotic illness. Likewise, a recent Australian national study (*n* = 185) [21] reported that 48.6% of autistic adults were in the clinical range for depression, and that 35.7% reported recent suicidal ideation. Hirvikoski et al. [14] showed that autism is associated with an increased risk of death by suicide. Using population-level data from Sweden, the authors reported that autistic people were 7.55 times more likely to die by suicide than were matched general population controls. Increased risk was observed for autism with comorbid intellectual disability (ID) (odds ratio [OR] = 2.41) as well as for autism without comorbid ID (OR = 9.40), and the risk was significantly higher in the latter group relative to the former. Autistic females (OR = 13.05) and autistic males (OR = 6.28) were at increased risk relative to females and males in the non-autistic population, respectively. However, the percentage of deaths by suicide was similar in these two groups (autistic females 0.32%; autistic males 0.30%). This suggests that the presence of an autism diagnosis is associated with a relatively greater increased risk of suicide in females than males. This is consistent with the findings of Kirby et al. [16], who recently reported that autistic females were at higher risk of dying by suicide than were non-autistic females (though see also [15]).

A considerable proportion of autistic people (both children and adults) remain undiagnosed or are misdiagnosed with other psychiatric conditions [22–28]. Some people remain undiagnosed or have been misdiagnosed with other conditions but suspect they may be autistic, whilst others do not recognise their symptoms [27]. Autism is usually considered in dichotomous terms (i.e. a person is thought to either be or not be autistic); however, variations in individual difference traits associated with autism are found to be normally distributed throughout the general population [29–31]. These ‘autistic traits’ can be measured quantitatively using the widely used, reliable, and valid Autism Spectrum Quotient (AQ) [29, 32]. A high score on the AQ may indicate the presence of undiagnosed autism, if the traits are interfering with a person's daily functioning [33]. The AQ consists of five subscales [29]: social skill, attention switching, attention to detail, communication, and imagination. Paquette-Smith et al. [18] reported significantly higher scores (indicating greater impairment) on the communication and attention switching subscales (as well as total AQ) in autistic adults who had attempted suicide compared to autistic adults who had not attempted suicide. Some similar concepts have also been investigated: for instance, Levi et al. [34] reported that communication difficulties (problems in self-disclosure) as well as the related constructs of schizoid traits, alexithymia, and loneliness were predictive of lethality of suicide attempts, and that the effects were significant even when psychiatric illness and mental pain (including depression and hopelessness) were controlled for.

There is some evidence to suggest an association between undiagnosed autism and suicidal behaviour. Takara and Kondo [27] reported that diagnosed or undiagnosed pervasive developmental disorder not otherwise specified (PDD-NOS) was more prevalent in adults with depression (major depressive disorder = 61%; bipolar disorder = 39%) who had attempted suicide compared to those who had not. The majority had not been diagnosed with autism at the time of their first clinical visit for current major depression, but 11% (including 29% of those who had attempted suicide) were eventually diagnosed during the study. Further evidence that undiagnosed autistic individuals may be at an increased risk of suicide comes from a recent study by Cassidy et al. [35], which analysed coroners’ records, from two regions in the UK, for which a conclusion (under older coronial law known as a ‘verdict’) of suicide or open was returned. Records were screened for evidence of autism, and the prevalence was estimated to be 11.3%. This was considerably higher than the ~ 1% of the UK general population that is diagnosed with autism, although it should be noted that for most cases identified, no diagnosis of autism by a medical professional was present.

The above evidence suggests that suicidal thoughts and behaviours may be more prevalent in autistic people compared to the general population. These effects appear to occur regardless of whether a diagnosis of autism has been made by a healthcare professional, suggesting that elevated autistic traits may be a marker of risk for suicidal behaviour. This has not been extensively tested, as most studies of suicidal behaviour have looked at autism as a dichotomous diagnostic category. However, if elevated autistic traits are associated with suicidal thoughts and behaviours, this could be a useful predictor of suicide risk whether or not an autism diagnosis is present. Similarly, variation in autistic traits in different domains (e.g. social difficulties, rigid thinking) in autistic people may help predict risk for suicidal behaviours.

There are several reasons why elevated autistic traits may be associated with suicidal thoughts and behaviour in people with or without a diagnosis of autism. First, individuals with elevated autistic traits experience more social difficulties, such as loneliness [21, 36], peer victimisation [37], and ‘camouflaging’ of autistic characteristics to fit in in social situations [38–41]. These are observable indicators of the state of ‘thwarted belonging’ (lack of meaningful social connections), which, according to the interpersonal theory of suicide, increases risk of contemplating suicide [42, 43]. Second, it has been speculated that cognitive inflexibility or ‘black and white’ thinking may increase suicidality by increasing the likelihood of becoming stuck in distressing and depressogenic thought patterns [44], which may reduce the ability to consider alternative courses of action than suicide. Such an interpretation is also consistent with predominant models of suicidal behaviour, such as the integrated motivational-volitional model of suicidal behaviour, which highlight entrapment as a proximal predictor of suicide [45–47]. Third, autistic traits are associated with reduced cognitive empathy or ‘theory of mind’ [48], which might make it more difficult for someone who feels suicidal to understand the emotional impact that their death may have upon their friends and family. Finally, autistic traits have been associated with increased vulnerability to depression and anxiety [21, 37, 49], which are strong markers for suicide risk [2, 4].

Some studies have looked at autistic traits and suicidality in samples of people diagnosed with autism. Cassidy et al. [11] and Paquette-Smith et al. [18] reported that higher scores on the AQ were associated with suicide plans or attempts in autistic adults. In contrast, Takara and Kondo [27] did not find evidence that higher AQ scores were associated with suicide attempts in autistic patients, and they did not test this relationship in non-autistic patients. Two studies by Hedley et al. [21, 50] reported that symptom severity (as measured by the AQ-Short, an abridged version of the AQ [51]) was not related to suicidal ideation in autistic adults. However, higher AQ-Short scores were independently predictive of higher levels of loneliness and depression, and lower levels of satisfaction with social support [21].

Recent studies have examined autistic traits in relation to suicidality in non-autistic populations. Pelton and Cassidy [52] found that AQ scores were positively correlated with lifetime suicidality in a young adult population. The authors reported that this relationship was significantly mediated by feelings of perceived burdensomeness and thwarted belongingness. In a sample of 381 neurotypical university students, Upthegrove et al. [53] reported that traits associated with autism (AQ) and psychosis were both predictive of depressive symptoms. These variables were also found to interact: AQ scores correlated more strongly with depressive symptoms when high levels of psychotic symptoms were present, and psychotic symptoms correlated more strongly with depressive symptoms when high AQ scores were present. In a second sample of 99 participants treated for first episode psychosis, AQ and current positive psychotic symptoms were both predictive of lifetime suicidality. A further analysis showed that 71% of the total association between AQ and lifetime suicidality was mediated by hopelessness. In another study [38] of 169 non-autistic adults who did not have first degree autistic relatives, AQ scores significantly predicted suicidality, after controlling for several relevant covariates: age, sex, employment, satisfaction with living arrangements, additional developmental conditions, depression, and anxiety. Hence, there are likely additional factors in the context of autism and autistic traits that increase the risk of suicidality beyond known risk markers in the general population.

The current study measured autistic traits in a population of adults who reported having made at least one suicide attempt. More specifically, we compared the level of autistic traits as measured by the AQ (total score as well as each subscale) between adults who had attempted suicide once and adults who had attempted suicide more than once. As noted above, the risk markers and the phenomenology of suicidal behaviour may be different in the context of autism. For example, high levels of autistic traits include reduced ability to switch attention, and increased repetitive behaviour, characteristics that might increase the risk of repeated suicide attempts. Along with the observation that previous research has reported elevated levels of autistic traits to be associated with higher prevalence/severity of suicidal thoughts and behaviours [38, 52, 53], we hypothesised that adults who had attempted suicide more than once would have higher total AQ scores and higher AQ subscale scores than adults who had attempted suicide once.

## Methods

### Participants

Of the 245 people that initially accessed the online survey, 168 (68.6%) completed the AQ. 181 (73.9%) participants answered whether they had attempted suicide; 141 had attempted suicide, and 40 had not. There were no differences between those who completed the AQ and those who did not in terms of their likelihood of having attempted suicide, *χ*^*2*^(1, 181) = 0.612, *P* = 0.434, *φ* = 0.058, or the number of times they had attempted suicide, *χ*^*2*^(1, 134) = 2.467, *P* = 0.116, *φ* = − 0.136.

The sample used for analysis included all participants who had both completed the AQ and specified that they had attempted suicide (*n* = 132). Of these, 17 (12.9%) were male, 110 (83.3%) were female, 1 (0.8%) was intersex, 1 (0.8%) was transsexual, 1 (0.8%) preferred not to say, and 2 (1.5%) responded with ‘other’. Of those who stated ‘other’, one specified that they were non-binary, and the other as genderqueer. Ages ranged from 18 to 65 (median = 36.00, interquartile range [IQR] = 19.00), although it should be noted that 34 participants did not report their age. Of the 132 participants who completed the AQ and confirmed that they had attempted suicide, 126 reported how many times they had attempted suicide (i.e. once or more than once), and 129 reported their age at first suicide attempt.

## Materials

### Demographic and life experience questionnaire

Participants were asked to state their age and sex (‘male’, ‘female’, ‘intersex’, ‘transsexual’, ‘prefer not to say’, ‘other’), as well as where they found the survey (e.g. having seen it advertised on a particular website or social media channel). They were then asked, “Have you ever attempted suicide?” (yes/no). If answering ‘yes’ to this question, participants were then asked, “How often have you attempted suicide” (‘once’ or ‘more than once’) and “How old in years were you when you first attempted suicide?” (answered via a dropdown menu, 1–99). The questions were adapted from those used by Cassidy et al. [11]. Participants were then asked to state any mental health and developmental conditions with which they had been diagnosed, and any they suspected they might have, even if they had not been formally diagnosed (see Table 1).

**Table 1:** Prevalence of mental health and developmental conditions in adults who had attempted suicide

	Diagnosed *n* (%)	Suspected *n* (%)
Alcohol/drug addictions and related disorders	12 (9.1)	8 (6.1)
Anxiety	75 (56.8)	21 (15.9)
Anorexia nervosa	19 (14.4)	2 (1.5)
Attention deficit and/or hyperactivity disorder	5 (3.8)	1 (0.8)
Autism spectrum condition	15 (11.4)	19 (14.4)
Bipolar disorder	9 (6.8)	6 (4.5)
Bulimia nervosa	9 (6.8)	1 (0.8)
Depression	103 (78.0)	15 (11.4)
Dyslexia	8 (6.1)	2 (1.5)
Dyspraxia	7 (5.3)	4 (3.0)
Epilepsy	4 (3.0)	0 (0.0)
Language delay	1 (0.8)	0 (0.0)
Learning difficulties	3 (2.3)	0 (0.0)
Obsessive compulsive disorder	14 (10.6)	8 (6.1)
Personality disorder	31 (23.5)	6 (4.5)
Schizophrenia	4 (3.0)	2 (1.5)
Tourette’s syndrome	1 (0.8)	0 (0.0)
Other	24 (18.2)	9 (6.8)

Data in this table relate to all participants who completed the AQ and confirmed that they had attempted suicide (*n* = 132)

### Autistic traits

We measured autistic traits with the Autism Spectrum Quotient [29] (AQ), a 50-item questionnaire that has been used widely in research settings. The AQ was developed to quantify autistic traits in people with average or above average intelligence. It consists of five subscales (social skill, attention switching, attention to detail, communication, imagination), which can be summed to create a total score. The instrument has good test-retest reliability [29] and can differentiate between autistic and neurotypical people [29, 33]. Although a score of ≥ 32 was initially suggested to indicate the likely presence of autism [29], Woodbury-Smith et al. [33] showed that a score of ≥ 26 provided good discriminant validity and screening properties in a clinically referred sample. Cronbach’s alpha (α) was calculated to examine internal consistency for the current study (i.e. for all participants who completed the AQ and reported that they had attempted suicide; *n* = 132). Internal consistency for the whole scale was excellent (α = 0.915); for the subscales, it was lower but still acceptable: social skill (α = 0.792), attention switching (α = 0.717), attention to detail (α = 0.668), communication (α = 0.807), and imagination (α = 0.718).

### Design and procedure

Before conducting the main study, a focus group was held (*n* = 3, all females) in which adults who reported having attempted suicide reviewed a draft version of the online questionnaire and provided feedback about how it felt for them to complete the survey. Additionally, two further adults (one female, one male) who reported having attempted suicide provided feedback via email. All participants gave written informed consent; their suggestions were reviewed by the research team, and relevant changes to the questionnaire were made. The final version was approved by the Psychology Research Ethics Committee, University of Cambridge (reference PRE.2016.084), and all procedures carried out were in accordance with the Declaration of Helsinki.

The current study utilised a cross-sectional design to examine whether autistic traits are associated with a history of suicide attempts. Adult participants who had attempted suicide were recruited to take part in an online survey. The study was primarily advertised on social media via an advert that asked people who had ever attempted suicide to consider completing an online survey to help mental health research. The survey was additionally advertised on mental health and suicide prevention websites, on a psychology research participant database, and on a suicide research participant database. All survey respondents accessed the online questionnaire between June and September 2017, and each provided informed consent prior to beginning the study.

Participants were provided with an information sheet, which gave details about the purpose of the study and the nature of the questions that would be included. They were advised that although most questions included in the survey would not be upsetting, some could be, and that they did not need to answer these questions if they did not want to. Informed consent was then required before participants could proceed to the questionnaire.

Due to the nature of the subject matter, as well as the population recruited, signposting to relevant services was provided prior to commencement of the study and again at the end of the debrief. These details were also made available as a download on each page of the questionnaire.

### Statistical analysis

Statistical analyses were performed using IBM SPSS version 24. AQ score and the five AQ subscales were treated as predictors of how many suicide attempts participants reported having made (one or more than one). All tests were two-tailed, and findings were considered statistically significant at *P* < 0.05.

Chi-square tests were used to compare the prevalence of diagnosed and suspected autism between adults who had attempted suicide once and adults who had attempted suicide more than once. This is because a difference in baseline autism prevalence between the two groups could potentially explain a score difference in autistic traits between the groups. AQ total scores were normally distributed, as indexed by Shapiro-Wilk test, though this was not the case for the subscales scores. For this reason, data for the subscales are presented as median and IQR. An independent samples *t* test was used to compare total AQ scores between adults who had attempted suicide once and adults who had attempted suicide more than once; non-parametric (Mann-Whitney *U*) tests were used to compare the AQ subscale scores. We then used a binary logistic regression analysis to further examine the association between AQ total score and number of suicide attempts whilst controlling for age, sex, and presence of mental health and developmental conditions that might be associated with elevated autistic traits (diagnosed or suspected anxiety, depression, anorexia nervosa, attention-deficit/hyperactivity disorder (ADHD), autism, and personality disorder).

## Results

### Description of the sample

Age at first suicide attempt ranged from 7 to 58 years (*n* = 129, median = 18.00, IQR = 9.00). Prevalence of (self-reported) diagnosed and suspected mental health and developmental conditions are reported in Table 1. Of the 17 males who completed the AQ and specified that they had attempted suicide, 4 (23.5%) reported an autism diagnosis, and 2 (11.8%) suspected that they were autistic. Of 110 females who completed the AQ and confirmed that they had attempted suicide, 11 (10.0%) reported a diagnosis of autism, and 16 (14.5%) suspected that they were autistic. Of five participants who could not be classified as either male or female, none reported an autism diagnosis, but one (20.0%) suspected they were autistic. This equates to 15 (11.4%) adults reporting an autism diagnosis and 19 (14.4%) reporting that they suspected they were autistic, who together comprised 25.8% of the total sample of 132. 35.3% of males, 24.5% of females, and 20.0% of those who could not be classified as male or female reported either diagnosed or suspected autism.

### Comparison of autistic traits between adults who had attempted suicide once and adults who had attempted suicide more than once

Of the adults that completed the AQ and reported that they had attempted suicide (*n* = 132), 126 reported whether they had attempted suicide once (*n* = 44) or more than once (*n* = 82). Before comparing AQ scores between these groups, we checked whether the prevalence of diagnosed and suspected autism differed. Adults with diagnosed autism were overrepresented in the group of participants who had made more than one suicide attempt, although the effect did not reach statistical significance, *χ*^*2*^(1, 126) = 3.491, *p* = 0.062, *φ* = − 0.166. The effect size would generally be considered small (small, *φ* = 0.1; medium, *φ* = 0.3; large, *φ* = 0.5) [54]. The effect for suspected autism was in the opposite direction and was not significant, *χ*^*2*^(1, 126) = 1.525, *p* = 0.217, *φ* = 0.110.

An independent samples *t* test (total AQ score) and Mann-Whitney *U* tests (AQ subscale scores) were used to compare autistic traits between adults who had attempted suicide once and adults who had attempted suicide more than once (Table 2). Adults who had attempted suicide more than once had significantly higher total AQ scores. According to generally accepted criteria [54], the effect size was small to medium (small, *d* = 0.2; medium, *d* = 0.5; large, *d* = 0.8). This group also had higher scores on each of the five subscales, though only the effects for communication and imagination were statistically significant. The effect sizes observed were small (small, *r* = 0.1; medium, *r* = 0.3; large, *r* = 0.5) [54].

**Table 2:** Total AQ and AQ subscale scores in adults who had attempted suicide once and adults who had attempted suicide more than once

	Overall sample			One attempt			More than one attempt						
	*N*	*M*	SD	*N*	*M*	SD	*N*	*M*	SD	*R*	DF	*P*	*D*
Total AQ	132	25.83	10.354	44	22.80	11.208	82	27.63	9.665	− 2.532	124	*0.013*	− 0.472
	*N*	Median	IQR	*N*	Median	IQR	*N*	Median	IQR	*U*	*Z*	*P*	*R*
Social skill	132	6.00	4.00	44	5.00	6.00	82	6.00	3.25	1548.500	− 1.316	0.188	− 0.117
Attention switching	132	7.00	3.00	44	6.00	4.00	82	7.00	4.00	1469.000	− 1.728	0.084	− 0.154
Attention to detail	132	5.50	3.00	44	5.00	4.00	82	6.00	4.00	1522.000	− 1.454	0.146	− 0.130
Communication	132	5.00	5.00	44	4.00	5.00	82	5.00	4.25	1346.000	− 2.356	*0.018*	− 0.210
Imagination	132	3.00	4.00	44	2.50	3.00	82	3.00	4.00	1297.000	− 2.617	*0.009*	− 0.233

Numbers in italics indicate effects considered statistically significant at the *P* < 0.05 level

To control for additional aspects of demography and presence of mental health and developmental conditions, we conducted a binary logistic regression analysis with number of suicide attempts (one or more than one) as the outcome variable, and total AQ score as the predictor. We controlled for sex (female, male, or other) and age (continuous), as well as reported diagnosed or suspected conditions that might be associated with relatively high levels of autistic traits. More specifically, we controlled for diagnosed/suspected autism, anxiety, anorexia, ADHD, depression, and personality disorder using dichotomous covariates (diagnosed/suspected or not diagnosed/not suspected). The regression model included *n* = 126, and female sex and absence of suspected/diagnosed conditions were set as the reference categories. The model fit was significant (Omnibus test of model coefficients: *χ*^*2*^ = 25.805, df = 10, *P* = 0.004); the model explained between 18.5% (Cox and Snell *R*^2^) and 25.5% (Nagelkerke *R*^2^) of variance in the outcome variable, and predicted 72.2% of cases correctly. There remained a trend towards significance for autistic traits (*P* = 0.052), with total AQ scores being higher in adults who had attempted suicide more than once compared to adults who had made one suicide attempt. There was also a significantly higher prevalence of diagnosed/suspected personality disorder in the group of adults who had attempted suicide more than once (Table 3)^1^.

**Table 3:** Binary logistic regression model with number of suicide attempts (one or more than one) as the outcome variable

	OR	95% CI	*P*
Total AQ	1.051	0.999, 1.105	0.052
Age	0.970	0.930, 1.011	0.150
Sex			0.206
Sex (male)	2.681	0.735, 9.777	0.135
Sex (other)	0.386	0.044, 3.417	0.392
Anorexia	3.091	0.770, 12.408	0.112
ADHD	3.060	0.299, 31.293	0.346
Anxiety	1.557	0.599, 4.045	0.364
Depression	0.907	0.262, 3.139	0.877
Personality disorder	4.032	1.343, 12.103	*0.013*
Autism	0.569	0.174, 1.861	0.351

Missing values for age (*n* = 34) were replaced with the median (36.00); numbers in italics indicate effects considered statistically significant at the *P* < 0.05 level

### Prevalence of elevated autistic traits in adults who had attempted suicide

To determine whether the elevated level of autistic traits in this sample may be explainable entirely by the high prevalence of diagnosed/suspected autism, all such participants (*n* = 34) were removed from the following analyses. The mean total AQ score for the remaining 98 participants was 22.45 (SD = 8.671). For reference, Ruzich et al. [30] reported a mean AQ total score of 16.94 based on a meta-analysis of 6934 non-clinical participants from 78 studies (reported in 73 articles).

Although the AQ is not intended as a diagnostic instrument, scores of ≥ 32 [29] and ≥ 26 [33] have been used to indicate clinical significance. Eighteen (18.2%) participants had a score of ≥ 32, and 40 (40.6%) had a score of ≥ 26 (note that if those who reported diagnosed or suspected autism are retained in the analysis, these are even higher: ≥ 32 = 44 [33.4%]; ≥ 26 = 71 [53.9%]). Of the five participants that could not be classified as male or female, three had AQ scores of ≥ 26, and two had AQ scores of ≥ 32 (individual AQ scores 14, 24, 27, 36, and 45).

## Discussion

The current study provides evidence that high levels of autistic traits (as measured by total AQ score) may often be present in adults who have attempted suicide. Even when autistic adults and adults who suspected they were autistic were removed from the analysis, 40.6% of those who had attempted suicide scored above the threshold (≥ 26) that indicates potential clinical concern. We also observed that adults who had attempted suicide more than once had elevated autistic traits compared to adults who had attempted suicide on one occasion. The findings broadly confirm those of previous studies reporting positive correlations between AQ scores and suicidality [11, 18, 38, 52, 53]. Screening for autism may be an important way to assess future risk in adults who are experiencing suicidal thoughts and behaviours.

Adults who had attempted suicide more than once had significantly higher total scores on the AQ than adults who had attempted suicide once, with a small to medium effect size being observed (*d* = − 0.472). In the multivariate analysis, we controlled not only for self-reported diagnoses of conditions that may be associated with elevated autistic traits (autism, ADHD, anxiety, depression, anorexia nervosa, and personality disorder), but also for suspected presence of these conditions. This is an important consideration because people with conditions such as borderline personality disorder [55] and anorexia nervosa [56, 57] have higher autistic traits than is typical of the general population. It has also been reported that comorbid populations with both autism and borderline personality disorder are at increased risk of suicide [26]. We found that the effect of total AQ score remained statistically significant (*P* = 0.028) in the analysis of participants who reported their age (*n* = 94), though it was only marginally significant (*P* = 0.052) when missing values for this variable were replaced with the median to allow for analysis of a larger sample size (*n* = 126). Taken together, the findings suggest that autistic traits may be associated with suicidal behaviour above and beyond established markers of risk in this population. This is consistent with recent findings that autism is an independent risk marker for suicide attempts whilst controlling for a range of demographic variables and comorbid conditions [58].

We also observed that adults who had attempted suicide more than once had significantly higher scores (indicating greater impairment) on the communication and imagination AQ subscales compared to individuals who had attempted suicide once. Although the effects for the other three subscales (social skill, attention switching, attention to detail) were not statistically significant, it is worth noting that they were all in the same direction (i.e. higher scores in adults who had attempted suicide more than once). These findings might suggest that suicidal behaviour is associated with the presence of autistic traits generally, though also perhaps with the presence of domain-specific aspects of an autistic personality style.

In the only other study so far to examine AQ subscale scores in relation to suicidality, Paquette-Smith et al. [18] found significantly higher attention switching and communication scores in autistic adults who had attempted suicide compared to autistic adults who had not attempted suicide. The authors suggested that communication impairments may increase difficulties in asking for help and that deficits in attention switching might increase rumination or impose additional challenges when coping with change. However, when comparing the results, it should be noted that the individuals who took part in the current study were recruited based on having made at least one suicide attempt, not because of their autism diagnostic status, and that they may represent a very specific subgroup of the population (i.e. people who did not die after making at least one suicide attempt). It is therefore difficult to infer from our findings whether they generalise to the wider population (and to autistic populations), or whether people who die at their first suicide attempt are also likely to exhibit high levels of autistic traits.

A key finding from the current study is that a very high prevalence of elevated autistic traits was observed in this sample of adults who reported having attempted suicide. Even when participants with diagnosed or suspected autism were removed from the analysis, 18.2% had an AQ score of ≥ 32, and 40.6% had a score of ≥ 26. As a score of ≥ 26 is considered a clinical screening cut-point that is used in adult diagnostic clinics in the UK [33], this implies that a considerable proportion of adults who have made suicide attempts may warrant a specialist diagnostic assessment to determine whether a diagnosis of autism is appropriate. This could potentially enable individuals to access additional support that could help improve aspects of their lives and help to prevent future suicide risk. This raises the question of whether adults who attempt suicide are more likely than the general population to have undiagnosed autism or to have been misdiagnosed with other psychiatric conditions. For example, one study reported that a considerable proportion of females diagnosed with borderline personality disorder may also meet the criteria for autism [26].

It is important to consider the implications of our results for general suicide theory, and why autistic traits are associated with attempting suicide. Autistic traits appear to increase vulnerability to experiencing risk markers for suicidal intent. For example, in the integrated motivational-volitional model of suicidal behaviour [45, 46], suicidal intent is precipitated by difficulties associated with high levels of autistic traits, such as in social problem solving [59], memory biases and ruminative processes [60], future thinking [61, 62], social support [50], thwarted belonging, and perceived burdensomeness [52]. As discussed above, the rigid thinking style characteristic of autism and associated with high levels of autistic traits could increase the risk of and/or amplify experiences of entrapment and thus suicidal intent. Given that entrapment is a key driver of suicidal behaviour in other high-risk populations, it is not surprising that individuals with high levels of autistic traits are at elevated risk, as many of these traits are likely to contribute to one’s sense of entrapment [46, 63]. However, future research must directly explore the associations between autistic traits, entrapment, and suicide attempts. Results from the current study also suggest that there may be aspects of suicidal behaviour unique to the case of high levels of autistic traits, which need to be accommodated for in current suicide theory, assessment tools, and prevention strategies.

## Limitations

There are several limitations to the current study. First, participants self-reported mental health and developmental conditions and history of suicidal behaviour, and such responses could not be independently verified. Additionally, single items were used to assess whether participants had ever attempted suicide (yes or no) and, if so, how many times they had attempted suicide (once or more than once) as well as their age at first suicide attempt. However, this approach is widely used in the research literature [4, 11, 17, 21].

The sample size was moderate. However, the inclusion criteria and nature of the topic made recruiting a large sample challenging, and other studies that have reported on populations of adults who have attempted suicide have been comparable in sample size. For example, although Takara and Kondo [27] presented data relating to 336 outpatients with major depressive disorder or bipolar disorder, only 31 were reported to have attempted suicide. Women were also over-represented in the current sample. This may reflect the observation that suicide attempts are more common in women than in men [3]. Another possibility is that women are more likely to respond to questionnaires of this nature. Evidence for this is provided in that some studies of suicidality in neurotypical adults have reported similar sex ratios (e.g. Upthegrove et al. [53] reported on a sample consisting of only 21% males). The relatively low number of males who took part in the current study was a limitation, as it meant that meaningful analyses of males and females separately could not be performed. Future research should aim to address this issue, particularly considering that males have higher levels of autistic traits compared to females [29, 30, 32], are more likely to be diagnosed with autism [25], and are more likely to die by suicide [2, 3]. This is further made clear by the observation that of the 17 males who completed the current survey and specified that they had attempted suicide, 6 (35.3%) were autistic or suspected they were autistic. However, it should not be overlooked that the risk of suicide might be disproportionately increased in autistic females relative to autistic males [14, 16].

When interpreting the finding that AQ scores appear to be elevated in the current study, it is important to consider that sampling bias could have played a role. This is because some participants would have taken part in the survey after having seen social media adverts from our research group. However, we controlled for this to some extent by removing from the relevant analysis those participants who were autistic or suspected they were autistic, and indeed the survey was also advertised by a number of other organisations that do not have specific links to autism. A further issue is that we did not record whether participants had first-degree autistic relatives, which is an important limitation considering that such people often have elevated levels of autistic traits [64]. However, even if the presence of such participants could explain the elevated levels of autistic traits observed in this sample as a whole, there is no reason to suspect that sampling bias would have affected the comparisons of autistic traits between adults who had attempted suicide once and adults who had attempted suicide more than once.

## Conclusions

The current study reports elevated autistic traits in adults who had attempted suicide, as well as higher levels of autistic traits in adults who had attempted suicide more than once compared to adults who had attempted suicide once. However, there remain several potential explanations for these findings. One possibility is that having elevated autistic traits is a direct predictor of suicidality. Another possibility is that undiagnosed autism is more prevalent in populations of adults who have attempted suicide than in the general population. Although we removed from analysis or statistically controlled for participants with diagnosed or suspected autism where appropriate, it cannot be discounted that some people may themselves be unaware of their undiagnosed autism. Another possibility is that autistic traits in people who have attempted suicide may be inflated due to the high prevalence of conditions such as borderline personality disorder and anorexia nervosa, which are themselves associated with elevated autistic traits. There is also a question regarding the direction of causality, as it is difficult to determine the extent to which states of mind produced in a suicidal crisis (likely present among those with or without elevated autistic traits) relate to antecedent personality traits. This is because aspects of the pre-suicidal mind state may resemble autistic traits (e.g. cognitive inflexibility, inability to consider alternative courses of action, reduced empathy), but can have different causes. Further research is urgently needed to disentangle the relationship between autistic traits and suicidality and to help us better understand what the risks and protective factors are and how to prevent future risk.

## Footnotes

1 The above analysis (*n* = 94) was rerun without substituting median age for missing values, and similar results were obtained. The model fit was significant (Omnibus test of model coefficients: *χ*^2^ = 23.876, df = 10, *P* = 0.008), though this time the level of autistic traits was a statistically significant predictor, with total AQ scores being higher in adults who had attempted suicide more than once compared to adults who had made one suicide attempt, OR = 1.068, 95% CI [1.007, 1.133], *P* = 0.028. The model explained between 22.4% (Cox and Snell *R*^2^) and 30.7% (Nagelkerke *R*^2^) of variance in the outcome variable, and correctly predicted 74.5% of cases.

## Abbreviations

ADHD: Attention deficit/hyperactivity disorder
AQ: Autism Spectrum Quotient
CI: Confidence interval
ID: Intellectual disability
IQR: Interquartile range
M: Mean
OR: Odds ratio
PDD-NOS: Pervasive developmental disorder not otherwise specified
SD: Standard deviation
